# *Listeria monocytogenes* MDR transporters are involved in LTA synthesis and triggering of innate immunity during infection

**DOI:** 10.3389/fcimb.2014.00016

**Published:** 2014-02-25

**Authors:** Keren Tadmor, Yair Pozniak, Tamar Burg Golani, Lior Lobel, Moran Brenner, Nadejda Sigal, Anat A. Herskovits

**Affiliations:** The Department of Molecular Microbiology and Biotechnology, The George S. Wise Life Sciences Faculty, Tel Aviv UniversityTel Aviv, Israel

**Keywords:** multidrug transporters, lipotechoic acid, c-di-AMP, type I interferon, *Listeria monocytogenes*

## Abstract

Multi-drug resistance (MDR) transporters are known eponymously for their ability to confer resistance to various antimicrobial drugs. However, it is likely that this is not their primary function and that MDR transporters evolved originally to play additional roles in bacterial physiology. In *Listeria monocytogenes* a set of MDR transporters was identified to mediate activation of innate immune responses during mammalian cell infection. This phenotype was shown to be dependent on c-di-AMP secretion, but the physiological processes underlying this phenomenon were not completely resolved. Here we describe a genetic approach taken to screen for *L. monocytogenes* genes or physiological pathways involved in MDR transporter-dependent triggering of the type I interferon response. We found that disruption of *L. monocytogenes* lipoteichoic acid (LTA) synthesis results in enhanced triggering of type I interferon responses in infected macrophage cells yet does not impact bacterial intracellular growth. This innate immune response required the MDR transporters and could be recapitulated by exposing macrophage cells to culture supernatants derived from LTA mutant bacteria. Notably, we found that the MDR transporters themselves are required for full production of LTA, an observation that links MDR transporters to LTA synthesis for the first time. In light of our findings, we propose that the MDR transporters play a role in regulating LTA synthesis, possibly via c-di-AMP efflux, a physiological function in cell wall maintenance that triggers the host innate immune system.

## Introduction

*Listeria monocytogenes* is a Gram-positive facultative intracellular pathogen and the causative agent of Listeriosis, a food born disease in humans (Swaminathan and Gerner-Smidt, 2007). *L. monocytogenes* invades phagocytic and non-phagocytic cells, the latter by using specialized surface proteins named internalins that actively trigger bacterial internalization (Hamon et al., 2006). Following internalization, the bacteria escape the initial vacuole/phagosome into the host cell cytosol via the action of several virulence factors, primarily the pore-forming hemolysin, Listeriolysin O (LLO) (Kathariou et al., 1987; Portnoy et al., 1988; Cossart et al., 1989; Smith et al., 1995; Rabinovich et al., 2012). Once in the cytosol, *L. monocytogenes* replicates and spreads from cell to cell by exploiting host actin filaments (Tilney and Portnoy, 1989). Mammalian cells specifically induce the type I interferon innate immune response in response to cytosolic growing *L. monocytogenes* (O'Riordan et al., 2002; Herskovits et al., 2007). This response is manifested by enhanced expression and secretion of the cytokine interferon-β (IFN-β) (O'Riordan et al., 2002), and involves several host innate immune signaling molecules such as STING, TBK-1, and IRF3 (Stockinger et al., 2004; O'Connell et al., 2005; Perry et al., 2005; Ishikawa et al., 2009; Jin et al., 2011; Sauer et al., 2011). On the bacterial side, several Multi-drug resistance (MDR) transporters were shown to be involved in triggering the type I interferon response via secretion of the cyclic di-nucleotide, c-di-AMP (Crimmins et al., 2008; Woodward et al., 2010; Yamamoto et al., 2012). In particular, two closely related MDR transporters, MdrM and MdrT, of the major facilitator superfamily (MFS), were identified in a genetic screen to promote induction of IFN-β when over expressed (Crimmins et al., 2008). While both transporters were shown to be involved in c-di-AMP secretion, only deletion of the *mdrM* gene resulted in a lower induction of IFN-β in infected cells, designating MdrM as the main MDR transporter that mediates this response. Taking together a model was proposed whereby during *L. monocytogenes* infection MdrM secretes c-di-AMP to the macrophage cytosol, where it is recognized by STING, an innate immune signaling adaptor, triggering activation of type I interferon response (Jin et al., 2011; Sauer et al., 2011). While these successive events were largely demonstrated, the preceding physiological conditions that explain why *L. monocytogenes* secretes c-di-AMP were not resolved.

Initially, c-di-AMP was discovered to regulate sporulation in response to DNA damage in *Bacillus subtilis* (Bejerano-Sagie et al., 2006; Witte et al., 2008). Subsequently, several reports have indicated additional roles for this unusual nucleotide in controlling bacterial cell size, cell wall stress, and peptidoglycan homeostasis in multiple species (Banerjee et al., 2010; Corrigan et al., 2011; Luo and Helmann, 2012; Kaplan Zeevi et al., 2013; Witte et al., 2013). Moreover, c-di-AMP is considered to play a role in fatty acid synthesis and growth under low potassium conditions in *Mycobacterium smegmatis* and *Staphylococcus aureus*, respectively (Corrigan et al., 2013; Zhang et al., 2013). Together these studies reveal that c-di-AMP is a key second messenger molecule that regulates multiple pathways in bacteria (Romling, 2008; Corrigan and Grundling, 2013). In *L. monocytogenes* c-di-AMP was shown to be essential for growth, extracellularly in broth and intracellularly in mammalian cells (Witte et al., 2013). It was identified in bacterial culture supernatants, primarily those derived from strains over expressing MdrM and MdrT, though the functional role of c-di-AMP secretion in *L. monocytogenes* remained largely unclear (Woodward et al., 2010; Yamamoto et al., 2012).

We have recently found that MdrM is not the only MDR transporter that contributes to IFN-β induction during *L. monocytogenes* infection. We identified a set of MDR transporters, homologs of MdrM, that together with MdrM are responsible for most of IFN-β induction (Kaplan Zeevi et al., 2013). These transporters, named MTAC transporters (for MdrM, MdrT, MdrA, and MdrC), are highly expressed during *L. monocytogenes* infection, yet are not necessary for intracellular growth in macrophage cells. An *in vitro* screen for physiological conditions that require the MTAC transporters revealed that they play a role in *L. monocytogenes* response to cell wall stress, specifically during inhibition of peptidoglycan synthesis. We found that a quadruple Δ*mdrMTAC* mutant is sensitive to vancomycin treatment due to an inability to produce and shed peptidoglycan, as a drug titration mechanism. Notably, over-degradation of c-di-AMP by over-expressing its phosphodiesterase PdeA increased the susceptibility of Δ*mdrMTAC* to vancomycin, whereas over-production of c-di-AMP via over-expression of its diadenylate cyclase DacA reduced the bacterial susceptibility to this drug. These results suggested that the MDR transporters together with c-di-AMP regulate cell wall maintenance, specifically during cell wall related stress conditions (Kaplan Zeevi et al., 2013).

In the present study we took an unbiased genetic approach to identify *L. monocytogenes* genes and/or pathways that trigger MdrM-dependent induction of type I interferons in infected macrophage cells. Remarkably, we found that aberrant LTA synthesis in *L. monocytogenes* triggered enhanced induction of IFN-β in an MDR-dependent manner. Moreover, we discovered that the MDR transporters themselves are required for LTA synthesis under normal growth conditions. This study demonstrates for the first time a link between MDR transporters and LTA synthesis, and provides further support to the premise that MDR transporters play a physiological role in bacterial cell wall regulation and synthesis.

## Results

### Genetic screen for *L. monocytogenes* genes that differentially modulate the MdrM-dependent type I interferon response

**Figure 1:**
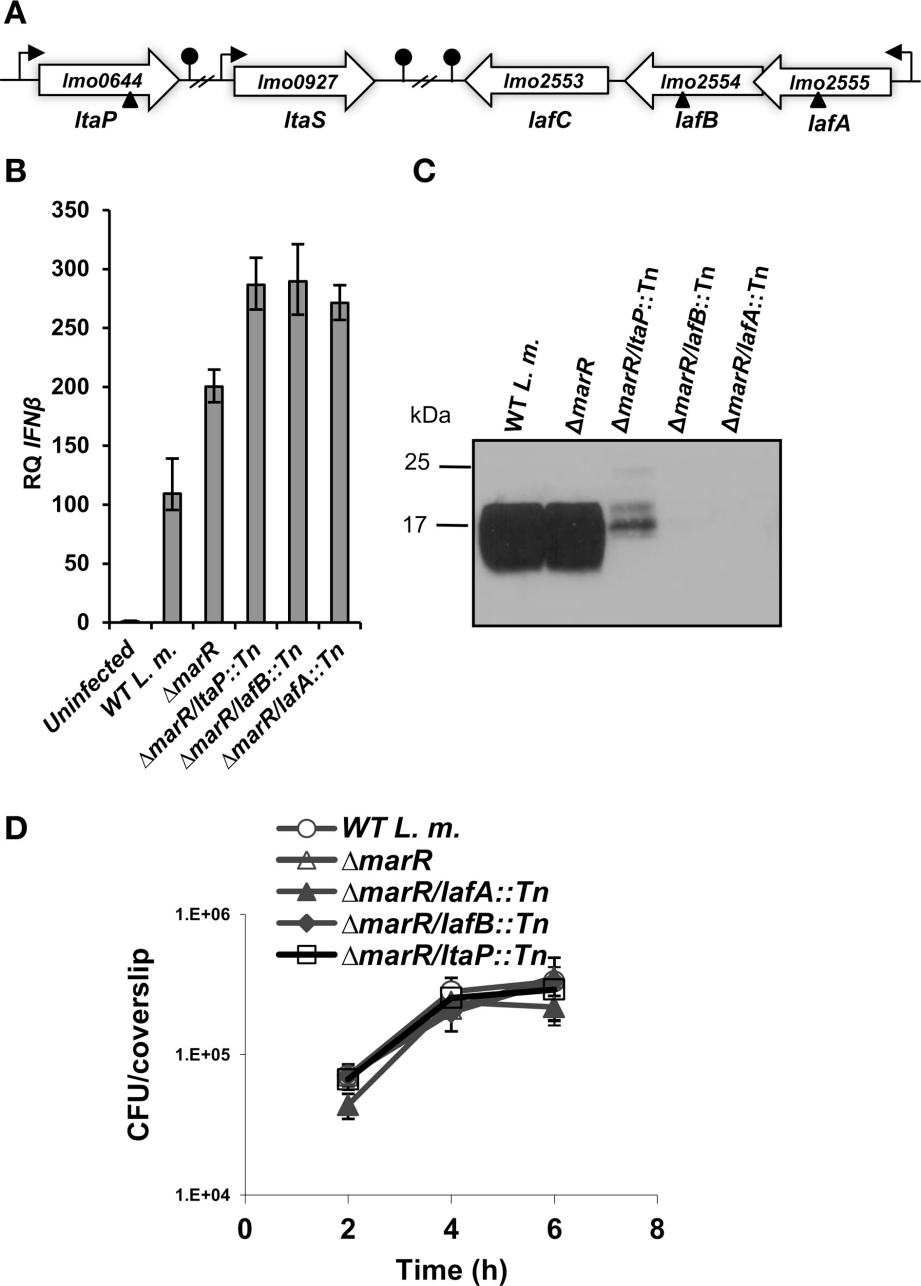
***L. monocytogenes* LTA mutants trigger enhanced IFN-β response. (A)** Schematic representation of the LTA biosynthesis genes in the *L. monocytogenes* 10403S genome. Transposon insertion sites are depicted by triangles. **(B)** RT-qPCR analysis of IFN-β transcription levels in BMD macrophage cells infected with WT *L. monocytogenes*, Δ*marR*, and LTA transposon mutants in the Δ*marR* background (Δ*marR/ltaP::Tn*, Δ*marR/lafB::Tn*, and Δ*marR/lafA::Tn*) at 6 h.p.i. Transcription levels are presented as relative quantity (RQ), relative to levels in uninfected cells. The data represent three biological repeats (*N* = 3). Error bars indicate 95% confidence interval (as described in Materials and Methods). **(C)** Western blot analysis of cell wall-associated LTA in WT, Δ*marR* and Δ*marR/ltaP::Tn*, Δ*marR/lafB::Tn* and Δ*marR/lafA::Tn* transposon mutants grown in BHI medium at 37°C using polyglycerolphosphate-specific LTA antibody (Clone 55, Hycult biotechnology). Twenty microgram of total protein were loaded onto SDS-PAGE 15% gel, as described in the Materials and Methods. **(D)** Intracellular growth curves of WT *L. monocytogenes*, Δ*marR*, and the LTA transposon mutants, in BMD macrophage cells. Representative growth curves are shown, one of three biological repeats (*N* = 3). Error bars represent the standard deviation of a triplicate.

As mentioned above, over-expression of MdrM results in enhanced triggering of type I interferon response in a manner that is dependent on c-di-AMP secretion (Woodward et al., 2010; Yamamoto et al., 2012). To better understand the physiological pathways involved in MdrM's function, we performed an unbiased genetic screen to identify *L. monocytogenes* genes that differentially modulate this MdrM-dependent type I interferon response. To achieve this goal a *L. monocytogenes Himar-mariner1* transposon library was generated in the background of the Δ*marR* strain (deleted of MdrM's repressor gene, *marR*), which highly expresses MdrM. As shown previously, this strain induces 2 to 3-fold higher levels of IFN-β in comparison to wild type (WT) bacteria upon infection of macrophage cells (Crimmins et al., 2008). Briefly, we used a previously described screening approach whereby IFN-β is measured in the supernatants of *L. monocytogenes*-infected cells by an ISRE-type I interferon reporter cell line that expresses luciferase in response to type I interferons (Jiang et al., 2005; Crimmins et al., 2008). Screening of ~6000 *L. monocytogenes* Δ*marR::marinerTn* mutants in 96-well format uncovered 12 and 16 low and high inducers of IFN-β, respectively, as compared to the Δ*marR* parental strain (Table 1). Among the low inducers, we identified mutants in genes encoding the flagellar system, the virulence master regulator PrfA and the LLO toxin, all factors that are known to mediate adhesion and invasion into host cells. We also identified genes related to cell wall and purine metabolism as well as unknown genes (Table 1A). Among the high inducers, we identified genes associated with lipoteichoic acid (LTA) biosynthesis, amino acid metabolism, cell division, transport and surface proteins (Table 1B). Notably, within this list, three genes were identified to be directly involved in LTA biosynthesis. LMRG_01692.6 (*lmo2555*) and LMRG_01693.6 (*lmo2554*) encode LTA anchor formation proteins, LafA and LafB respectively, and are both cytoplasmic glycosyltransferases involved in the synthesis of the LTA glycolipid anchor Gal(α1-2)Glc(α1-3)-diacylglycerol (Gal-Glc-DAG). The third LTA biosynthesis gene, LMRG_00310.2 (*lmo0644*), encodes the LTA primase, LtaP that transfers the first glycerolphosphate group onto the Gal-Glc-DAG anchor (Figure 1A) (Webb et al., 2009). Since these genes represent the first three sequential and critical steps of the LTA biosynthesis pathway, we decided to further investigate their role in triggering of the type I interferon response.

**Table 1:** ***L. monocytogenes* mutants that differentially activate IFN-β**.

**Insertion site**	***L. m.* EGDe gene identifier**	**Description**	**Pathway**
**(A) *L. monocytogenes* MUTANTS THAT INDUCE LOW LEVELS OF IFN-β**			
LMRG_02622.6	*lmo0200*	*prfA*, main virulence regulator of *L. monocytogenes*	Virulence
LMRG_02624	*lmo0202*	*hly*, encoding Listeriolysin O toxin	Virulence
LMRG_00367.6	*lmo0679*	Similar to flagellar biosynthetic protein flhB	Mobility and chemotaxis
LMRG_00380.6	*lmo0692*	Two-component sensor histidine kinase CheA	Sensor/signal transduction
LMRG_00386.2	*lmo0697*	Similar to flagellar hook protein FlgE	Mobility and chemotaxis
LMRG_00394	*lmo0705*	Similar to flagellar hook-associated protein FlgK	Mobility and chemotaxis
LMRG_00397.6	*lmo0708*	Similar to hypothetical flagellar protein	Mobility and chemotaxis
LMRG_00402.2	*lmo0713*	Flagellar basal-body M-ring protein fliF	Mobility and chemotaxis
LMRG_02761.6	*lmo1687*	N/A	Unknown
LMRG_02767.6	*lmo1693*	N/A	Unknown
LMRG_01002.6	*lmo1855*	D-alanyl-D-alanine carboxipeptidase	Cell wall
LMRG_01003.6	*lmo1856*	Purine nucleoside phosphorylase	Nucleotide metabolism
**(B) *L. monocytogenes* MUTANTS THAT INDUCE HIGH LEVELS OF IFN-β**			
LMRG_00151.6	*lmo2750*	Similar to para-aminobenzoate synthase component I	Amino acids metabolism
LMRG_00277.6	*lmo0595*	Similar to O-acetylhomoserine sulfhydrylase	Amino acids metabolism
LMRG_00310.2	*lmo0644*	Membrane sulfatase family protein	LTA
LMRG_00332.2	*lmo2469*	Similar to amino acid transporter/amino acid permease family protein	Transporters
LMRG_00541.6	*lmo1079*	Similar to YfhO	Unknown
LMRG_00672.6	*lmo1226*	Similar to transporter (*B. subtilis* YdgH); lmo1225 is a transcriptional regulator from marR family	Transporters
LMRG_00860.2	*lmo1408*	*ladR*, a transcriptional repressor for *mdrL*	Regulation
LMRG_01641.6	*lmo2191* (~180 upstream)	Aresnate reductase, transcriptional regulator spx family	Regulation
LMRG_01692.6	*lmo2555*	Glycosyl transferase	LTA
LMRG_01693.6	*lmo2554*	Galactosyl transferase	LTA
LMRG_01775.6	*lmo2473*	N/A	unknown
LMRG_01983.6	*lmo2713*	Cell wall bound protein, contains 1 GW-repeat	Cell surface proteins
LMRG_02415.6	*lmo0170*	Hypothetical protein	Unknown
LMRG_02642.6	*lmo0220*	Highly similar to cell division protein *ftsH*	Cell division
LMRG_02673.6	*lmo0233*	Similar to DNA repair protein *radA/sms* from *E. coli*	DNA repair
LMRG_00225.6/02811.6	*lmo1786* (~500 bp upstream)	Insertion is a 100 bp upstream of an undefined 36 aa ORF	Unknown

First, to validate the high-inducer phenotype, bone marrow derived (BMD) macrophage cells were infected with each of the LTA mutants, Δ*marR/lafA::Tn*, Δ*marR/lafB::Tn*, Δ*marR/ltaP::Tn*, and real time quantitative PCR (RT-qPCR) analysis of IFN-β expression was performed at 6 h post infection (h.p.i.). As shown in Figure 1B, all the three mutants triggered higher levels of IFN-β relative to the parental strain Δ*marR* and WT bacteria. Next, we examined whether these mutants are indeed defective in LTA synthesis. The mutants were grown in brain heart infusion (BHI) medium, in which each grew like WT bacteria (data not shown), and their LTA was extracted at mid-exponential phase as previously described (Webb et al., 2009). LTA was visualized via Western blot analysis using a polyglycerolphosphate-specific antibody (Webb et al., 2009). In line with previous studies (Webb et al., 2009), bacteria-bearing transposons in the *lafA* or *lafB* genes exhibited severely reduced LTA synthesis, whereas bacteria bearing a transposon in the *ltaP* gene displayed a reduced and modified LTA production (Figure 1C). This analysis established that the transposon mutants are indeed deficient in LTA biosynthesis. Next, the ability of the LTA mutants to grow intracellularly in BMD macrophages was evaluated. Remarkably, we observed that although the mutants hardly produce LTA, they grow efficiently in macrophage cells like WT bacteria (Figure 1D).

### LTA is dispensable for *L. monocytogenes* growth, yet its deficient synthesis leads to enhanced triggering of type I interferon response

**Figure 2:**
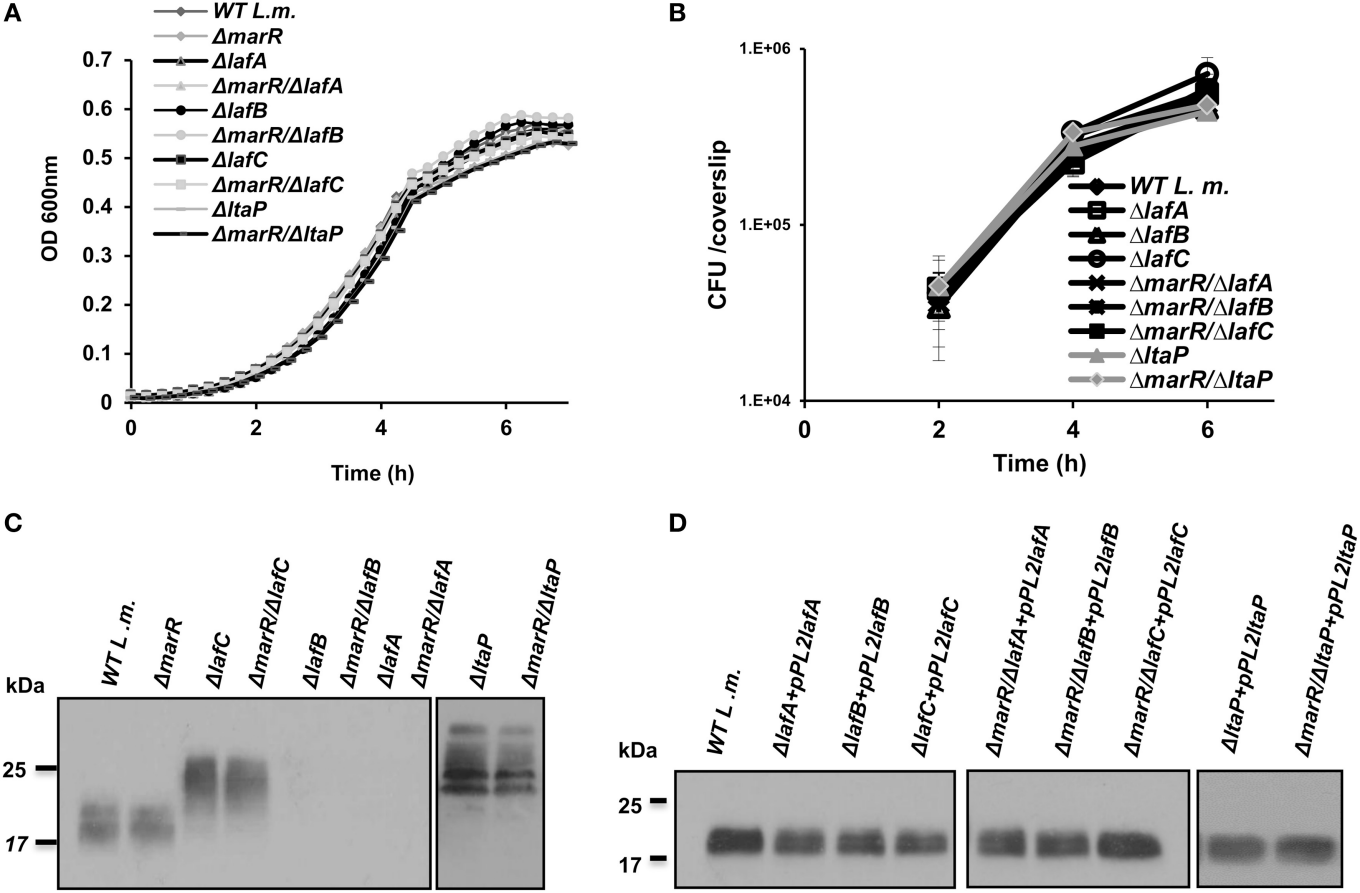
**LTA is dispensable for *L. monocytogenes* growth in rich laboratory medium and intracellularly in macrophage cells. (A)** Growth curves of WT *L. monocytogenes*, Δ*marR* and in-frame deletion mutants of *lafA*, *lafB*, *lafC*, and *ltaP* genes in the background of WT and Δ*marR* bacteria in BHI medium at 37°C. Experiment was performed in a 96-well format in a Synergy HT Biotek® plate reader. Error bars representing standard deviation of a triplicate are hidden by the symbols. The data is a mean of three biological repeats (*N* = 3). **(B)** Intracellular growth curves of WT *L. monocytogenes*, Δ*marR* and in-frame deletion mutants of *lafA*, *lafB*, *lafC*, and *ltaP* gens in the background of WT and Δ*marR* bacteria in BMD macrophage cells. Representative growth curves are shown, one of three biological repeats (*N* = 3). Error bars represent standard deviation of a triplicate. **(C)** Western blot analysis of cell wall-associated LTA derived from WT, Δ*marR*, Δ*lafC*, Δ*lafB*, Δ*lafA*, and Δ*ltaP* in the background of WT and Δ*marR* bacteria grown in BHI at 37°C. A polyglycerolphosphate-specific antibody was used (Clone 55, Hycult biotechnology). Five microgram of total protein were loaded onto SDS-PAGE 15% gel. **(D)** Western blot analysis of cell wall-associated LTA derived from LTA mutant strains complemented with their corresponding gene on the pPL2 integrative plasmid and grown in BHI at 37°C (10 μg of total protein were loaded onto SDS-PAGE 15% gel).

To further investigate the impact of aberrant LTA synthesis on triggering of the host innate immune system, a series of in-frame deletion mutants were generated in *lafA, lafB*, and *ltaP* genes, as well as in the *lafC* gene that was also shown to be involved in LTA production (Figure 1A) (Webb et al., 2009). Gene deletions were generated in the background of both WT and Δ*marR* bacteria (Table 2). As demonstrated in Figure 2, all mutants grew similarly to WT bacteria in BHI medium and intracellularly in BMD macrophage cells (Figures 2A,B). Nevertheless, the deletion mutants exhibited an LTA profile similar to that described by Webb et al. (2009), namely the *lafC* and *ltaP* mutants exhibited high-molecular weight LTA structures detectable by Western blots using a polyglycerolphosphate-specific antibody, whereas *lafA* and *lafB* mutants hardly produced detectable LTA (Figure 2C). Of note, introducing a copy of *lafA, lafB, lafC*, and *ltaP* genes on the integrative plasmid pPL2 (resulting in pPL2*lafA*, pPL2*lafB*, pPL2*lafC*, and pPL2*ltaP*) (Table 2) completely restored LTA production in the corresponding LTA mutants and in the Δ*marR* isogenic strains (Figure 2D). Taken together, these data validate the previously described roles of *lafA*, *lafB*, *lafC*, and *ltaP* genes in LTA synthesis, but more importantly demonstrate that LTA is dispensable for *L. monocytogenes* extracellular growth in broth and intracellular growth in mammalian cells. These findings were somewhat surprising, as LTA is known to be an important polymer in the cell wall of Gram-positive bacteria and therefore expected to influence bacterial virulence (Weidenmaier and Peschel, 2008; Reichmann and Grundling, 2011).

**Table 2:** **List of bacterial strains used in this study**.

**Strain**	**Genotype**	**References**
***Listeria monocytogenes***		
10403S	Wild type, Str r (WT)	Portnoy, DA lab stock
Δ*marR*	10403S Δ*marR* (LMRG_01348.6, lmo1618)	Crimmins et al., 2008
Δ*marR/lafA*::*Tn*	Δ*marR/lafA*::*Tn*	This study
Δ*marR/lafB*::*Tn*	Δ*marR/lafB*::*Tn*	This study
Δ*marR/ltaP*::*Tn*	Δ*marR/ltaP*::*Tn*	This study
Δ*lafA*	10403S Δ*lafA* (LMRG_01692.6, lmo2555)	This study and Webb et al., 2009
Δ*lafB*	10403S Δ*lafB* (LMRG_01693.6, lmo2554)	This study and Webb et al., 2009
Δ*lafC*	10403S Δ*lafC* (LMRG_01694.6, lmo2553)	This study and Webb et al., 2009
Δ*ltaP*	10403S Δ*ltaP* (LMRG_00310.2, lmo0644)	This study and Webb et al., 2009
Δ*marR*/Δ*lafA*	10403S Δ*lafA* (LMRG_01692.6, lmo2555) Δ*marR* (LMRG_01348.6, lmo1618)	This study
Δ*marR*/Δ*lafB*	10403S Δ*lafB* (LMRG_01693.6, lmo2554) Δ*marR* (LMRG_01348.6, lmo1618)	This study
Δ*marR*/Δ*lafC*	10403S Δ*lafC* (LMRG_01694.6, lmo2553) Δ*marR* (LMRG_01348.6, lmo1618)	This study
Δ*marR*/Δ*ltaP*	10403S Δ*ltaP* (LMRG_00310.2, lmo0644) Δ*marR* (LMRG_01348.6, lmo1618)	This study
Δ*mdrM*	10403S Δ*mdrM* (LMRG_02976.6, lmo1617)	Crimmins et al., 2008
Δ*mdrMTA*	*10403S* Δ*mdrM* Δ*mdrT* (LMRG_02679.6, lmo2588) Δ*mdrA* (LMRG_00200.6, lmo0519)	Kaplan Zeevi et al., 2013
Δ*mdrMTAC*	*10403S* Δ*mdrM* Δ*mdrT* Δ*mdrA* Δ*mdrC* (LMRG_01880.6, lmo2818)	Kaplan Zeevi et al., 2013
Δ*mdrM*/Δ*lafA*	*10403S* Δ*mdrM* Δ*lafA*	This study
Δ*mdrMTA*/Δ*lafA*	10403S Δ*mdrM* Δ*mdrT* Δ*mdrA* Δ*lafA*	This study
Δ*mdrMTAC*/Δ*lafA*	10403S Δ*mdrM* Δ*mdrT* Δ*mdrA* Δ*mdrC* Δ*lafA*	This study
Δ*mdrMTAC*/Δ*lafB*	10403S Δ*mdrM* Δ*mdrT* Δ*mdrA* Δ*mdrC* Δ*lafB*	This study
Δ*mdrMTAC*/Δ*lafC*	10403S Δ*mdrM* Δ*mdrT* Δ*mdrA* Δ*mdrC* Δ*lafC*	This study
Δ*mdrMTAC*/Δ*ltaP*	10403S Δ*mdrM* Δ*mdrT* Δ*mdrA* Δ*mdrC* Δ*ltaP*	This study
Δ*lafA*+*pPL2lafA*	10403S Δ*lafA* harboring the integrative plasmid pPL2 encoding *lafA* gene under its native promoter	This study
Δ*marR*/Δ*lafA*+*pPL2 lafA*	10403S Δ*marR*/Δ*lafA* harboring the integrative plasmid pPL2 encoding *lafA* gene under its native promoter	This study
Δ*lafB*+*pPL2 lafB*	10403S Δ*lafB* harboring the integrative plasmid pPL2 encoding *lafB* gene under its native promoter	This study
Δ*marR*/Δ*lafB*+*pPL2 lafB*	10403S Δ*marR*/Δ*lafB* harboring the integrative plasmid pPL2 encoding *lafB* gene under its native promoter	This study
Δ*lafC*+*pPL2lafC*	10403S Δ*lafC* harboring the integrative plasmid pPL2 encoding *lafC* gene under its native promoter	This study
Δ*marR*/Δ*lafC*+*pPL2lafC*	10403S Δ*marR*/Δ*lafC* harboring the integrative plasmid pPL2 encoding *lafC* gene under its native promoter	This study
Δ*ltaP*+*pPL2ltaP*	10403S Δ*ltaP* harboring the integrative plasmid pPL2 encoding *ltaP* gene under its native promoter	This study
Δ*marR*/Δ*ltaP*+*pPL2ltaP*	10403S Δ*marR*/Δ*ltaP* harboring the integrative plasmid pPL2 encoding *ltaP* gene under its native promoter	This study
***Escherichia coli***		
XL-1b	*recA1 endA1 gyrA96 thi-1 hsdR17 supE44 relA1 lac* [F' *proAB lacIqZ*Δ*M15 Tn10* (*Tetr*)].	Stratagene
SM-10	Conjugation donor; F-*thi*-1 *thr*-1 *leuB6 recA tonA21 lacY1 supE44 (Muc*^+^) λ-[*RP4-2*(*Tc::Mu*)] *Kmr Tra*^+^	Simon et al., 1983

**Figure 3:**
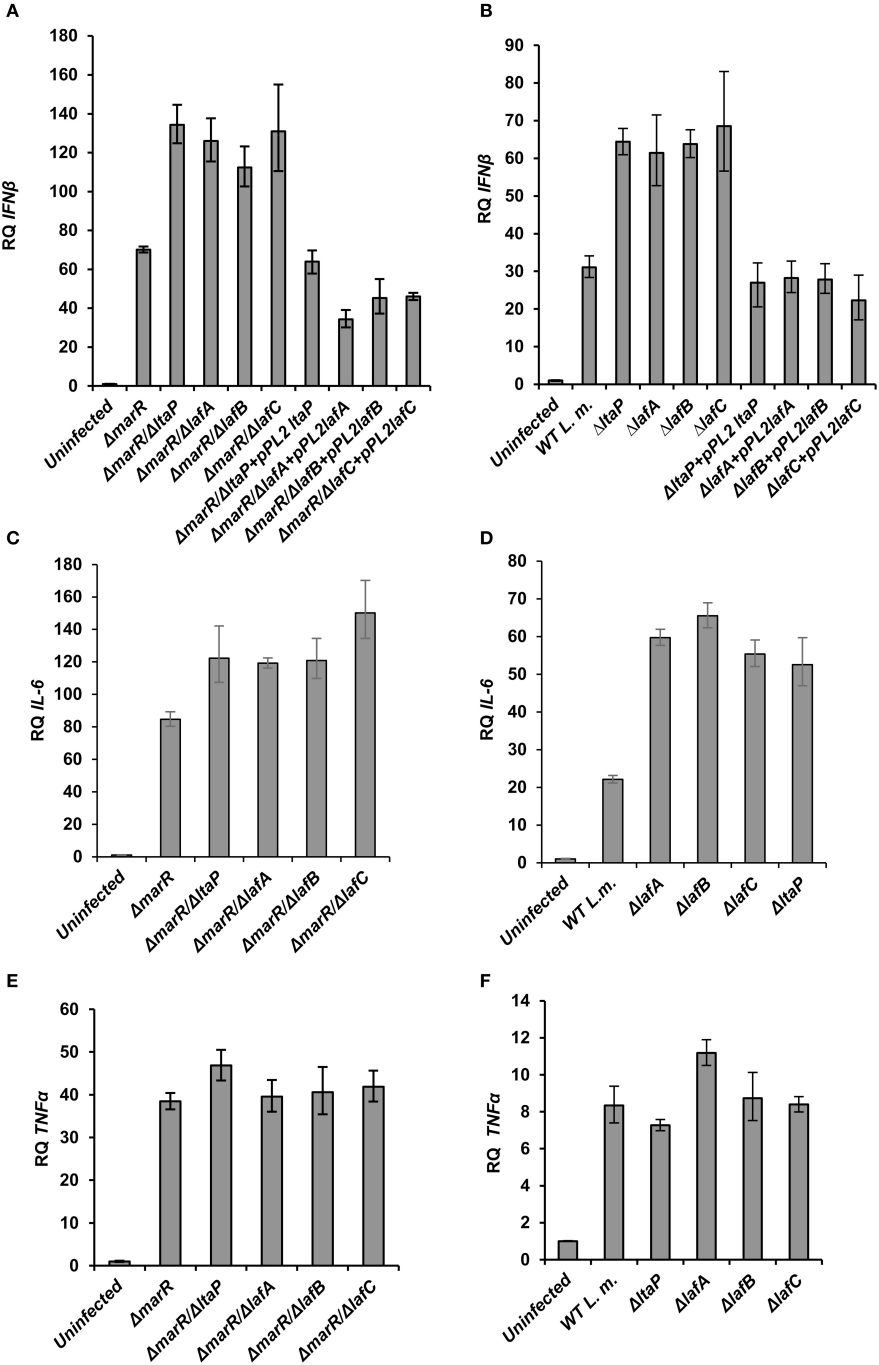
**LTA mutants in both WT and Δ*marR L. monocytogenes* background trigger an enhanced type I interferon response in macrophage cells.** RT-qPCR analysis of IFN-β, IL-6, and TNF-α (**A–D;** and **E,F**, respectively) 6 h.p.i. of BMD macrophage cells with the following bacterial strains: WT, Δ*marR*, and strains carrying deleted LTA genes (Δ*ltaP*, Δ*lafA*, Δ*lafB*, Δ*lafC*) in the background of WT or Δ*marR* and corresponding complemented strains. Transcription levels are represented as relative quantity (RQ), relative to the levels in uninfected cells. The data represent three biological repeats (*N* = 3). Error bars indicate 95% confidence interval, as described in the Materials and Methods (*P* < 0.01).

To gain further insight into the innate immune response elicited upon infection with the various LTA mutants, BMD macrophage cells were infected with Δ*lafA*, Δ*lafB*, Δ*lafC*, and Δ*ltaP* mutants in WT or the Δ*marR* background, and IFN-β, IL-6 (which is also part of the type I interferon response to *L. monocytogenes* infection) and TNF-α transcript levels were analyzed at 6 h.p.i. using RT-qPCR. As shown in Figure 3, each LTA mutant triggered increased level of IFN-β and IL-6 in comparison to his parental strain, without any detectable effect on TNF-α transcription (Figures 3A–E). Further, IFN-β induction by the LTA mutants could be restored by complementing their respective genes on a pPL2 plasmid (Figures 3A,B). Notably, the transcription levels of IFN-β and IL-6 were greater in cells infected with the Δ*marR*-LTA mutants than in cells infected with the LTA mutants in a WT background (~2-fold). These results demonstrate that although deletion of each LTA biosynthesis gene is sufficient to trigger an enhanced type I interferon response, an additive effect is observed when combined with the Δ*marR* deletion (which leads to over-expression of MdrM). Overall the data supports that aberrant LTA synthesis in *L. monocytogenes* triggers activation of the type I interferon response and not of other pro-inflammatory responses, such as those mediated by TNF-α. It is surprising that although LTA is a known immuno-stimulatory pathogen-associated microbial pattern (PAMP) (Takeuchi and Akira, 2001; Draing et al., 2008), we found that reduced bacterial LTA synthesis is associated with enhanced activation of the innate immune system. One possible explanation for this host-pathogen phenotype is that aberrant production of LTA triggers a bacterial response which, in turn, triggers activation of innate immune signaling pathways.

### The induction of IFN-β by LTA mutants is dependent on MDR transporters

**Figure 4:**
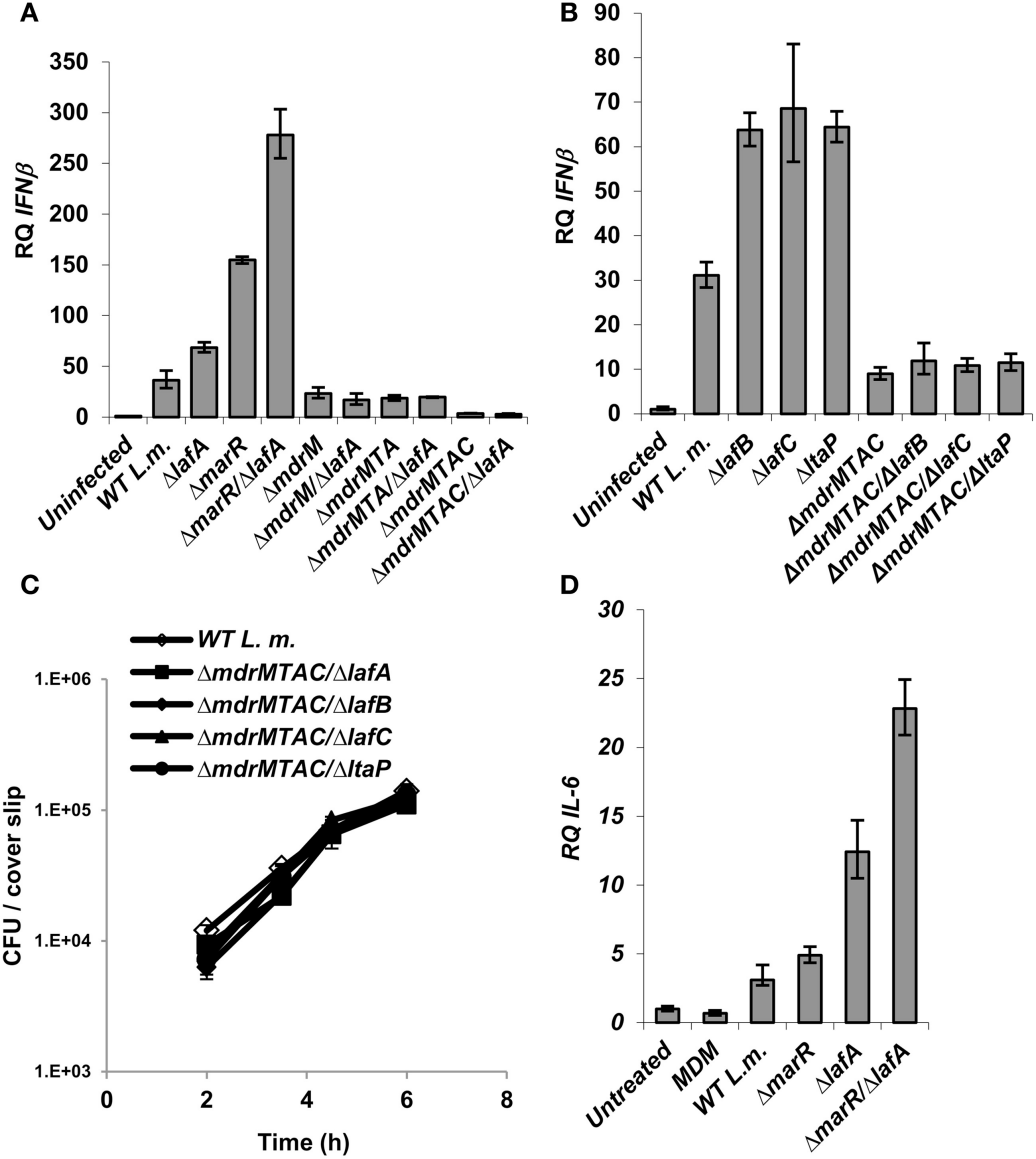
**Activation of IFN-β by *L. monocytogenes* LTA mutants requires the MDR transporters. (A)** RT-qPCR analysis of IFN-β transcription levels in BMD macrophage cells 6 h.p.i. with the following *L. monocytogenes* strains: WT, Δ*marR*, Δ*lafA*, and Δ*lafA* in combination with MDR deletions (Δ*mdrM*, Δ*mdrMTA*, and Δ*mdrMTAC)* in the background of WT or Δ*marR*. Transcription levels are represented as relative quantity (RQ), relative to uninfected cells. The data represents 3 biological repeats. Error bars indicate 95% confidence interval, *P* < 0.01 (as described in Materials and Methods). **(B)** RT-qPCR analysis of IFN-β transcription levels in BMD macrophage cells 6 h.p.i. infected with the following *L. monocytogenes* strains: WT and Δ*lafB*, Δ*lafC*, Δ*ltaP* in the background of WT or Δ*mdrMTAC*. Transcription levels are represented as relative quantity (RQ), relative to uninfected cells. The data represents 3 biological repeats. Error bars represent 95% confidence interval, *P* < 0.01. **(C)** Intracellular growth curves of WT *L. monocytogenes* and LTA mutants (Δ*lafA*, Δ*lafB*, Δ*lafC*, and Δ*ltaP*) in the background of the Δ*mdrMTAC* strain, in BMD macrophage cells. Representative growth curves are shown, one of three biological repeats (*N* = 3). Error bars represent the standard deviation of a triplicate. **(D)** RT-qPCR analysis of IL-6 transcription levels in BMD macrophage cells exposed to culture supernatants derived from the following *L. monocytogenes* strains: WT, Δ*marR*, Δ*lafA*, and Δ*marR*/Δ*lafA*. Bacteria were grown in minimal defined media (MDM) at 37°C and supernatants collected as described in the Materials and Methods and added to BMD macrophages for 6 h. A representative experiment is shown. The experiment was performed in three independent biological repeats. Transcription levels are represented as relative quantity (RQ), relative to untreated cells. Error bars indicate 95% confidence interval (*P* < 0.01).

Since we observed that aberrant LTA synthesis and MdrM over-expression each trigger enhanced type I interferon responses (and trigger additively in combination), we examined if each induce IFN-β independently or converge on the same mechanism. To this end, Δ*lafA* was combined with several MDR deletion mutants, Δ*mdrM*, Δ*mdrMTA* (a triple mutant of *mdrM*, *mdrT*, and *mdrA* genes), and Δ*mdrMTAC* (a quadruple mutant of *mdrM*, *mdrT*, *mdrA*, and *mdrC* genes), which were shown to mediate most of the IFN-β response to *L. monocytogenes* infection (Kaplan Zeevi et al., 2013). BMD macrophage cells were infected with the combination mutants, Δ*mdrM*/Δ*lafA*, Δ*mdrMTA*/Δ*lafA*, and Δ*mdrMTAC*/Δ*lafA* (Table 2) and with the respective parental mutant strains and WT bacteria, and IFN-β transcript levels were analyzed at 6 h.p.i. As shown in Figure 4A, the enhanced induction of IFN-β by the Δ*lafA* mutant was completely dependent on the MDR transporters, as it was abolished in the background of the MDR deletion mutants. The data indicates that deletion of *mdrM* alone is enough to reduce most of the IFN-β response triggered by the Δ*lafA* mutant, though deletion of more MDR genes reduced this response further (Kaplan Zeevi et al., 2013). Similar results were obtained with Δ*lafB*, Δ*lafC*, and Δ*ltaP* mutants, as shown in Figure 4B in combination with the quadruple Δ*mdrMTAC* mutant. Notably, these combined mutants hardly trigger IFN-β though grow normally in macrophage cells like WT bacteria (Figure 4C). Overall, these results suggest that the MDR transporters mediate the enhanced immuno-stimulatory phenotype of the LTA mutants.

Since these MDR transporters are considered to facilitate secretion of immuno-stimulatory molecules, as shown for c-di-AMP, we investigated if deficient LTA production triggers enhanced secretion of such molecules. To examine this hypothesis, the immuno-stimulatory potency of Δ*lafA* and WT bacteria culture supernatants were analyzed. Briefly, bacteria were grown to mid-exponential phase in minimal-defined medium (MDM), their supernatants were collected and added to BMD macrophage cells and IL-6 transcription levels were analyzed 6 h later. In line with our hypothesis, the supernatant derived from the Δ*lafA* mutant activated a higher IL-6 response as compared to that derived from WT bacteria (Figure 4D). Similarly, the supernatant of the double mutant Δ*marR*/Δ*lafA* was even more immune-stimulatory than that of Δ*marR* or Δ*lafA* alone (Figure 4D). Together these data support the model that defective LTA production leads to secretion of immune-stimulatory bacterial moieties that can be sensed by the innate immune system. Unfortunately, at this point we could not confirm nor exclude c-di-AMP as the secreted molecule.

### The MDR transporters are involved in LTA production

**Figure 5:**
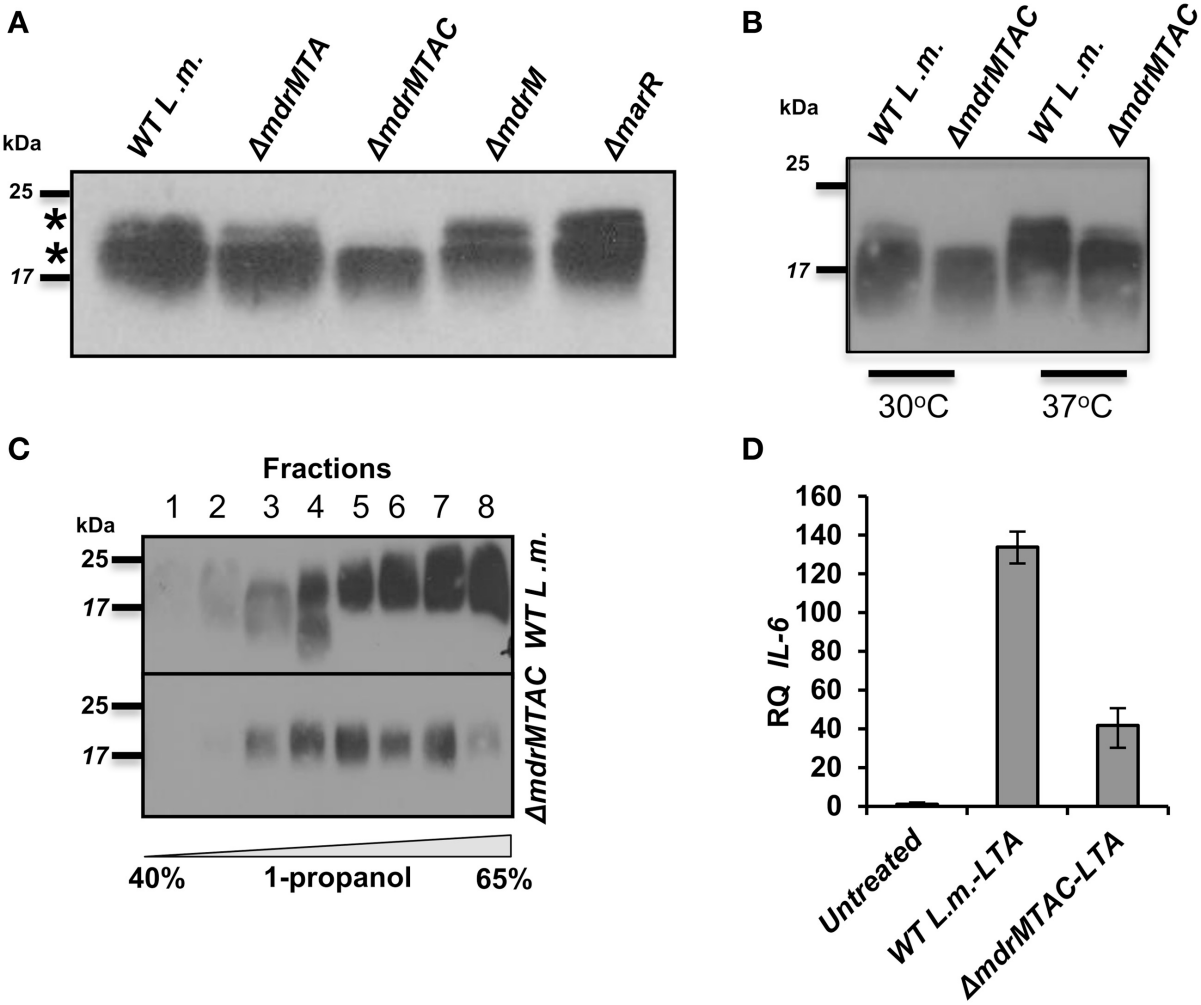
**Deletion of multiple *L. monocytogenes* MDR transporters interferes with normal LTA biosynthesis. (A)** Western blot analysis of cell-wall associated LTA derived from WT *L. monocytogenes*, Δ*mdrMTA*, Δ*mdrMTAC*, Δ*mdrM*, and Δ*marR* grown in BHI at 37°C. Five microgram of total protein were loaded onto SDS-PAGE 15%. LTA was detected using polyglycerolphosphate-specific antibody. **(B)** Western blot analysis of cell-wall associated LTA derived from WT *L. monocytogenes* and Δ*mdrMTAC* bacteria grown in BHI medium at both 30 and 37°C; 5 μg of total protein were loaded onto SDS-PAGE 15%. LTA was detected using polyglycerolphosphate-specific antibody. **(C)** Western blot analysis of HPLC fractions of cell-wall associated LTA derived from WT *L. monocytogenes* and Δ*mdrMTAC* bacteria grown in BHI medium at 37°C. LTA was extracted by water/butanol extraction method and separated on HPLC using analytical RP-C8 column. Gradient represents percentage of ammonium acetate-propanol (15–65%). LTA was obtained in the range of 40–65% 1-propanol. The data is representative of three biological repeats. **(D)** RT-qPCR analysis of IL-6 transcription levels in BMD macrophage cells exposed for 6 h to LTA derived from WT *L. monocytogenes* and Δ*mdrMTAC*. LTA was extracted using water/butanol. Hundred microgram of total protein were added to two million macrophage cells. Representative experiment is shown. The experiment was performed in three independent biological repeats. Transcription levels are represented as relative quantity (RQ), relative to untreated cells. Error bars indicate 95% confidence interval (*P* < 0.01).

To further explore the relationship between LTA synthesis and MDR transporters, we performed an independent experiment that evaluated LTA production in the various MDR deletion mutants. To this end, Δ*mdrM*, Δ*mdrMTA*, and Δ*mdrMTAC* mutants were grown to mid-exponential phase in BHI medium at 37°C, and their LTAs were extracted and compared with those of Δ*marR* and WT bacteria using Western blot analysis. Remarkably, we found that while most of the mutants exhibited an LTA profile similar to that of WT bacteria, Δ*mdrMTAC* mutant exhibited an altered profile, specifically missing a high molecular weight LTA band in the gel (Figure 5A). Careful examination of the LTA Western blots indicated that indeed two separate LTA bands appear (marked with asterisks), one that is thin with a high molecular weight and the other in many cases much thicker slightly lower in the gel. These bands, which can be easily missed if the LTA samples are overloaded, potentially represent different LTA variants.

In this regard, distinct LTA variants harboring different structures have been reported previously for *L. monocytogenes* strains (Hether and Jackson, 1983; Uchikawa et al., 1986; Dehus et al., 2011). For example, a recent report by Dehus at el. demonstrated a growth temperature-dependent expression of two LTA structural variants, named LTA1 and LTA2 (Dehus et al., 2011). LTA1 was shown to be produced at both 30°C and 37°C and comprises the canonical LTA structure. In contrast, LTA2 was shown to be produced predominantly at 37°C and harbors two diacyl-glycerol moieties, instead of one, linked to the Gal-Glc disaccharide (Dehus et al., 2011). To characterize in more detail the LTA deficiency associated with MDR transporters, WT and Δ*mdrMTAC* bacteria were grown at 30 or 37°C temperatures and their LTAs were extracted and analyzed. As shown in Figure 5B, WT bacteria grown at 37°C exhibited a more intense upper LTA band in comparison to bacteria grown at 30°C, in accordance with LTA2 production. Notably, Δ*mdrMTAC* mutant was completely missing the upper LTA band at 30°C, and demonstrated a weak band at 37°C in comparison to WT bacteria. These results suggest that the MTAC transporters contribute to the production of this LTA variant, which appears to correspond to LTA2. Of note, WT and Δ*mdrMTAC* bacteria exhibited comparable levels of the lower LTA variant.

To better delineate between the two LTA variants, the LTAs of WT and Δ*mdrMTAC* bacteria (grown at 37°C) were further isolated using a water/butanol extraction method and then separated by reverse phase HPLC using an analytic C8 column (Morath et al., 2001; Draing et al., 2006). Western blot analysis of the HPLC fractions clearly demonstrated that in the Δ*mdrMTAC* mutant one LTA variant is observed across the HPLC fractions. In contrast, WT bacteria produce several variants differing in molecular weight, which are distributed throughout the fractions (Figure 5C). Taken together, these results demonstrate that the Δ*mdrMTAC* mutant exhibits an altered LTA profile in comparison to WT bacteria, largely missing one or more distinct LTA variants.

Lastly, we assessed the immuno-stimulatory potency of the LTA of Δ*mdrMTAC* vs. WT bacteria. Bacteria were grown to mid-exponential phase in BHI at 37°C and LTAs were extracted using water/butanol as described previously (Morath et al., 2001; Draing et al., 2006). Purified LTAs were then added to BMD macrophage cells, and IL-6 transcription levels were analyzed using RT-qPCR. Of note, IL-6 was utilized as the readout since *L. monocytogenes* cell wall preparations do not induce much IFN-β when added to macrophage cells extracellularly (unpublished data). As shown in Figure 5D, the LTA of Δ*mdrMTAC* mutant was much less immuno-stimulatory than the LTA of WT bacteria, demonstrating that Δ*mdrMTAC* LTA is not only different in its composition but also in its ability to activate the innate immune system. Importantly, for the first time these observations establish a relationship between MDR transporters and LTA synthesis and furthermore, implicate a role for MDR transporters in LTA production/regulation in bacteria.

## Discussion

In this study we took an unbiased approach to investigate the *L. monocytogenes* genes or physiological pathways that trigger an MDR-dependent induction of type I interferon response during mammalian cells infection. A genetic screen was devised to identify *L. monocytogenes* genes that trigger induction of IFN-β during infection of macrophage cells. In this way, two groups of mutants were identified as high and low inducers of IFN-β. Among the low inducer mutants we identified genes that are necessary for invasion and intracellular growth such as, *hly* (encoding LLO), *prfA*, flagella and purine metabolism. Such mutations result in fewer bacteria per cell, which likely explain the reduced triggering of the type I interferon response relative to WT bacteria. Among the high inducer mutants, we identified diverse genes related to amino acid metabolism, cell division and DNA repair, with the LTA biosynthesis pathway conspicuously over represented in this group. Three independent mutants were identified as harboring a transposon inserted in distinct LTA synthesis genes, highlighting this bacterial pathway as a potential stimulator of the innate immune system. Though LTA itself is a known ligand recognized by receptors of the innate immune system, such as TLR2 (Takeuchi and Akira, 2001; Draing et al., 2008), here we demonstrate, a related yet seemingly converse phenotype, that perturbed LTA synthesis in bacteria can lead to enhanced activation of innate immune responses.

LTA is an important polymer of the cell wall of Gram-positive bacteria. Together with peptidoglycan and wall teichoic acids (WTA), it forms an extracellular envelope that serves the bacteria as a scaffold for binding of surface proteins and also protects from lysis (Weidenmaier and Peschel, 2008). LTA is an anionic polymer consisting of poly-glycerolphosphate chains that are linked to the membrane via a glycolipid anchor (Fischer et al., 1990). It is decorated with D-alanyl and glycosyl residues, which are substituted with glycerolphosphate groups (Fischer et al., 1980; Fischer, 1988). Such D-alanylation of LTA was shown to play a major role in bacterial resistance to cationic antimicrobial peptides, due to the increased positive charge provided by the D-alanyl residues (Baddiley, 2000). In *L. monocytogenes*, the genes responsible for LTA synthesis were only recently identified (Webb et al., 2009) and include two glycosyltransferases, *lafA* and *lafB* that sequentially bind glucose and galactose to a DAG moiety, and two distinct enzymes, LtaP and LtaS, that subsequently transfer glycerolphosphate groups (taken from phosphatidylglycerol) to the Gal-Glc-DAG anchor. LtaP was suggested to function as an LTA primase that transfers the first glycerolphosphate group to the Gal-Glc-DAG, whereas LtaS functions as an LTA synthase that extends the poly-glycerolphosphate chain via repetitive addition of glycerolphosphate units (Webb et al., 2009). Among these core LTA biosynthesis genes, only *ltaS* was shown to be required for *L. monocytogenes* growth *in vitro*, whereas the other LTA genes were found to be dispensable. This difference in the LTA mutant phenotypes is not completely understood, with one possibility being that the Δ*ltaS* mutant accumulates a toxic precursor or molecule that leads to growth inhibition.

In our screen we identified three out of the four LTA biosynthesis genes, excluding *ltaS*, in accordance with its documented lethal phenotype. Although these genes have been characterized to some extent, the role of LTA in *L. monocytogenes* infection and virulence has not been directly investigated. A previous study demonstrated that LTA D-alanylation is important for *L. monocytogenes* adhesion and invasion into mammalian cells (Abachin et al., 2002). Notwithstanding, mutants in the Dlt operon, which catalyzes the incorporation of D-alanines into the LTA, were shown to harbor increased LTA electronegativity, which in and of itself could underlie the observed phenotypes. To the best of our knowledge, the present study is the first to directly examine the role of LTA in *L. monocytogenes* infection of mammalian cells and to find it dispensable. All the LTA mutants generated in this study demonstrated normal intracellular growth in macrophage cells and also extracellularly, in both rich and minimal laboratory media. Some Gram-positive bacteria were shown to require LTA for growth (Grundling and Schneewind, 2007; Garufi et al., 2012; Richter et al., 2013). In this regard we speculate that *L. monocytogenes* might compensates for the lack of LTA by producing other polymers or alternatively by modifying existing ones, such as peptidoglycan and WTA (Corrigan et al., 2011).

**Figure 6:**
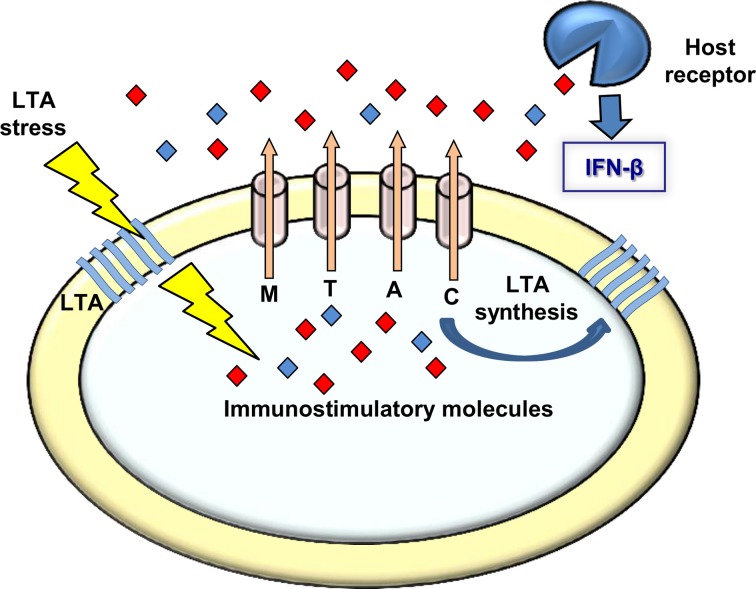
**A model linking MDRs, LTA, and induction of type I interferon response.** Upon LTA stress *L. monocytogenes* bacteria secret immuno-stimulatory molecules via the MDR transporters. These molecules are recognized by receptors of the innate immune system leading to type I interferon response. Under normal growth conditions the MDR transporters are involved in LTA synthesis, possibly via secretion of the same immuno-stimulatory molecules that might serve the bacteria as second messenger regulatory molecules regulating LTA synthesis.

Whether *L. monocytogenes* LTA-mutants produce other polymers or modify those that exist, it is clear that a bacterial response is initiated upon perturbation of LTA synthesis that triggers activation of type I interferons during infection. Our observations that bacteria bearing defects in LTA synthesis trigger an enhanced MdrM-dependent type I interferon response (also MTAC-dependent), a response that is even bigger when MdrM is over-expressed (i.e., in the Δ*marR* background), strongly suggest that MDR transporters are involved in bacterial management of LTA stress. Furthermore, the finding that this enhancement of the type I interferon response can be recapitulated by exposing macrophages to culture supernatants derived from LTA mutants, supports the premise that the MDR transporters mediate secretion of immuno-stimulatory molecules under conditions of LTA stress (Figure 6). In this regard, it was already evidenced that MdrM secretes the immuno-stimulatory molecule c-di-AMP under normal conditions, and that in turn, this molecule is capable of activating the type I interferon response (Woodward et al., 2010; Jin et al., 2011; Sauer et al., 2011).

Unfortunately, we could not verify nor exclude the involvement of c-di-AMP in the *L. monocytogenes* response to LTA stress nor in the enhanced induction of IFN-β by bacteria defective in LTA synthesis. However, a recent study by Corrigan at el. demonstrated a functional association between c-di-AMP and LTA stress in *Staphylococcus aureus* (Corrigan et al., 2011). In this study an *S. aureus ltaS* mutant was constructed, which does not produce LTA nor grow under laboratory conditions. Under this background, compensatory mutations were screened that allow bacteria to grow and a suppressor mutant in c-di-AMP phosphodiesterase gene (*gdpP*) was identified. Notably, this mutant exhibited increased levels of intracellular c-di-AMP, suggesting a role for this second messenger molecule in overcoming LTA stress. The authors further demonstrated that c-di-AMP leads to up-regulation of peptidoglycan cross-linking, as a way to compensate for the lack of LTA (Corrigan et al., 2011). In accordance with these findings, we speculate that also *L. monocytogenes* responds to LTA perturbation by producing c-di-AMP, which in this case is further secreted via the MDR transporters. Such a model would explain why the *L. monocytogenes* LTA mutants induce high levels of IFN-β in an MDR-dependent manner (Figure 6). Secretion of c-di-AMP is yet to be demonstrated in *S. aureus*, but we consider it likely that both species use a similar mechanism to overcome LTA stress.

Having identified an association between LTA synthesis and MDR transporters in the context of type I interferon induction, we further investigated this novel relationship under normal laboratory growth conditions. Specifically we asked whether the MDR transporters play a role in LTA synthesis, and not only during LTA stress responses. Surprisingly, we found that the MDR transporters, particularly the four MTAC transporters, are required for production of one or more distinct LTA variants. Previous studies have indicated that the MTAC transporters exhibit a functional redundancy, which could explain why this LTA phenotype was observed only in the quadruple mutant (Kaplan Zeevi et al., 2013). Interestingly, the MTAC transporters influenced production of an LTA variant that behaved like a previously described temperature dependent structural variant, named LTA2 (Dehus et al., 2011). LTA2 exhibits an unusual and rare structure harboring two diacyl-glycerol groups instead of one, and is primarily expressed at 37°C, which is the temperature of mammalian cells. Whether the MTAC-dependent LTA variant is indeed LTA2 needs to be determined biochemically, nevertheless the data indicates that the MTAC transporters play a role in the production of different LTA variants that could be expressed during mammalian cell infection. Moreover, we demonstrated that LTA derived from Δ*mdrMTAC* is much less immuno-stimulatory than LTA derived from WT bacteria. This observation supports that the MTAC-dependent LTAs are recognized by the innate immune system, which further suggests that the remarkable low immuno-stimulatory phenotype of the Δ*mdrMTAC* mutant could be partially due to the lack of these LTAs (Kaplan Zeevi et al., 2013).

In summary, this study establishes a functional association between LTA synthesis and MDR transporters, which is demonstrated both during macrophage cell infection and *in vitro* under laboratory conditions. Together with our previous findings that the MTAC transporters also play a role during peptidoglycan stress, we propose a model whereby bacterial MDR transporters play a global role in cell wall regulation and maintenance under diverse growth conditions and stresses. We hypothesize that the MDR transporters fulfill this function via secretion of secondary signaling molecules that regulate downstream bacterial cell wall responses. We anticipate that additional, yet to be discovered, signaling molecules take part in this complex regulation and not only c-di-AMP. Further studies are required to validate that c-di-AMP indeed plays a role in *L. monocytogenes* LTA stress, and to delineate the identities of down-stream molecular targets and regulated genes.

## Materials and methods

### Bacterial strains, cells, growth media, and reagents

*L. monocytogenes* 10403S strain was used as WT strain and as parental strain for all mutants generated in this work (Table 2). *E. coli* XL-1 blue strain (Stratagene) was used for vector propagation. *E. coli* SM-10 strain (Simon et al., 1983) was used for conjugative plasmid delivery to *L. monocytogenes* bacteria. *L. monocytogenes* strains were grown in BHI (Merck^©^) medium or MDM (Phan-Thanh and Gormon, 1997) at 37°C and *E. coli* strains were grown in Luria Bertani (LB, Acumedia^©^) medium at 37°C. For infection experiments *L. monocytogenes* bacteria were grown overnight in BHI at 30°C without agitation. Primary BMD macrophages were isolated from 6 to 8 weeks old female C57BL/6 mice (Harlan Laboratories Ltd, Israel) and cultured as described (Portnoy et al., 1989). ISRE-L929 reporter cell line was used to evaluate the levels of IFN-β secreted to the supernatants of infected macrophage cells (a gift from Bruce Butler, University of Texas Southwestern Medical Center) (Jiang et al., 2005).

### Genetic screen for *L. monocytogenes* mutants the differentially induce IFN-β

*L. monocytogenes* library of *Mariner1-Tn* mutants was constructed as described in (Zemansky et al., 2009) using 10403S Δ*marR Listeria monocytogenes* mutant as a background strain. Screening was performed as described in Crimmins et al. (2008). Briefly, a total of 6000 mutants were grown in BHI medium in 96-well plates overnight at 30°C. BMD macrophages from C57BL/6 mice were plated in 96-well plates, 4 × 10^4^ macrophages per well. Macrophages in each well were infected with 2 × 10^6^ bacterial cells. Thirty minutes post-infection, macrophages were washed and gentamicin was added (50 μg/ml) to kill extracellular bacteria. At 6 h post infection (h.p.i.), macrophage culture supernatants were taken and frozen at −80°C. The amount of IFN-β in the culture supernatants was detected using the type I interferon reporter cell line (ISRE-L929) (Jiang et al., 2005). Reporter cells were grown in 96-well plates and incubated with 10× dilution of the macrophage culture supernatants for 4 h. Cells were lysed using Glo Lysis Buffer (Promega) and luciferase activity was detected using the Beetlejuice D-Luciferine kit (PJK) and measured in a luminescence plate reader (Biotek Synergy HT). Transposon insertion sites were identified as described in Zemansky et al. (2009). Gene deletion (in frame) mutants were generated by standard techniques using pKSV7oriT derivate vector (pBHE261) (Lauer et al., 2008).

### *L. monocytogenes* infection and growth

Intracellular *L. monocytogenes* growth was performed as described previously (Lobel et al., 2012). Briefly, 2 × 10^6^ BMD macrophage cells were seeded on a petri dish with glass cover slips and infected with 8 × 10^6^ bacteria. At 0.5 h.p.i. cells were washed and at 1 h.p.i. gentamicin (50 μg/ml) was added. At each time point, cells from 3 cover slips were lyzed, diluted, and plated on BHI agar plates. Bacterial CFUs were counted after overnight growth at 37°C. For extracellular growth, overnight *L. monocytogenes* cultures were adjusted to OD_600nm_ of 0.02 in 200 μl of fresh BHI medium and were further grown in a Synergy HT Biotek® plate reader at 37°C with continuous shaking.

### LTA extraction and detection by western BLOT

LTA extraction was modified from a previously described method by Webb et al. (2009). Overnight cultures were adjusted to OD_600nm_ of 0.05 in 20 ml of BHI medium. Cultures were grown to OD_600nm_ of 0.5 then washed with Buffer-A (20 mM Tris-HCl pH-8, 0.5 M NaCl, and 1 mM EDTA) and resuspended with 1 ml of the same buffer. Samples were vortexed with 0.5 ml glass beads 425–600 μm (Sigma Aldrich) for 45 min, and then centrifuged for 1 min at 200 g to pellet the beads. Five hundred microliter of the supernatant were pelleted by centrifugation at 20,000 g for 15 min, at 4°C. The supernatants were discarded and the pellets were resuspended in 50 μl of Buffer-A with 2% SDS. The samples were boiled for 20 min and centrifuged at high-speed for 1 min. The supernatants were analyzed for total protein content by Lowry assay, and samples with equal amounts of total proteins were separated in a 15% SDS-polyacrylamide gel and probed with polyglycerolphosphate-specific LTA antibody (Clone 55, Hycult biotechnology) and HRP-conjugated goat anti-mouse IgG (Jackson ImmunoResearch, USA) used at 1:1000 and 1:20,000 dilutions, respectively. Western blots were developed by enhanced chemiluminescence reaction (ECL).

### Water/butanol LTA extraction

For further analysis, LTA was purified by water/butanol extraction protocol as described previously (Morath et al., 2001; Draing et al., 2006). In brief, bacteria cultivated overnight were adjusted to OD_600nm_ 0.1 in 200 ml of BHI medium, and grown to OD_600nm_ of 1 at 37°C. Bacterial cultures were then kept on ice for 45 min, and washed with 0.1 M sodium citrate (pH = 4.7). Bacterial pellets were suspended in 20 ml of the same buffer and lysed by French-Press (Stansted Fluid Power, UK) at 16,000 psi. Bacterial lysates were centrifuged at 17,000 g for 20 min and suspended in 10 ml of 0.1 M sodium citrate and in an equal amount of 1-butanol (Merck). Samples were stirred for 30 min and then centrifuged at 17,000 g for 20 min to achieve a two-phase separation. The lower aqueous phase was collected and a fresh phase of 10 ml sodium citrate 0.1 M was added. Extraction was repeated two more times, yielding a total of 30 ml of LTA extract. Samples were then evaporated by rotary evaporator, suspended in 10 ml of water and lyophilized.

### LTA reverse phase HPLC separation

Ten milligram of dry LTA was resuspended in 0.5 ml of chromatography start buffer (15% 1-propanol (Sigma Aldrich) in 0.1 M ammonium acetate, pH = 4.7) and centrifuged at 27,000 g for 60 min. The supernatant was subjected to reverse phase high-performance liquid chromatography (RP-HPLC) (JASCO) on a C8 analytic column (Symmetry C8, 5 μm, 3.9 × 150 mm, Waters) using a linear gradient of 15–60% of 1-propanol in 0.1 M ammonium acetate (pH = 4.7). Two milliliter fractions were collected lyophilized and resuspended again for Western blot analysis. The protocol was slightly modified from (Lehner et al., 2001; Grundling and Schneewind, 2007)

### Preparations of bacterial supernatants

Overnight cultures were inoculated in a 20 ml of MDM (Phan-Thanh and Gormon, 1997) at OD_600nm_ 0.02. Cultures were grown at 37°C to OD_600nm_ 0.5, and then were centrifuged at 3800 rpm for 20 min at 4°C. Supernatants were filtered through a 0.2 μm filter device and stored at −80°C. 2 ml of supernatants were lyophilized and resuspended in 100 μl of sterile water from which 50 μl were added to BMD macrophage cells for 6 h.

### Quantitative real time PCR analysis

RNA from infected macrophages or macrophages treated with bacterial supernatants or LTA extracts was obtained using Trizol reagent according to standard protocols. One microgram (1 μg) of RNA was reverse transcribed to cDNA using High Capacity reverse transcription kit® (Applied Biosystems). RT-qPCR was performed on 10 ng of cDNA using SYBER Green® with Step-one Plus RT-PCR system (Applied Biosystems). The transcription level of macrophage cytokines was normalized to the reference gene GAPDH. Statistical analysis was performed using the StepOne™ V2.1 software. Error bars represent a 95% confidence interval, i.e., the value is expected to fall within the bar range in 95% of repeat experiments. When the error bars of two samples do not overlap, the significance of the difference (*p*-value) is <<0.01.

### Conflict of interest statement

The authors declare that the research was conducted in the absence of any commercial or financial relationships that could be construed as a potential conflict of interest.
